# Molecular Design of Underwater Adhesive Copolymers: Synergy Between Long-Chain Alkyl Crystallization–Melting Switching and Carboxyl Group Interfacial Interactions

**DOI:** 10.3390/ma19112407

**Published:** 2026-06-05

**Authors:** Han Liu, Lei Hou

**Affiliations:** College of Chemistry and Chemical Engineering, Donghua University, Shanghai 201620, China; houlei@dhu.edu.cn

**Keywords:** underwater adhesion, cystallization-melting switching, hydrophobic interaction, Hydrogen bonding

## Abstract

Achieving strong adhesion in underwater or humid environments remains challenging because the interfacial hydration layer prevents direct contact between the adhesive and the substrate. Conventional adhesives typically fail under these conditions, so new strategies are needed to actively displace the water layer and create stable interfacial interactions. In this study, we prepared a series of copolymers with different monomer ratios via photocuring, using methacrylic acid (MAA) and stearyl methacrylate (SMA) as monomers. We focused on their thermal transition behavior and adhesion performance under both dry and underwater conditions. The results show that at an SMA molar fraction of 85%, the copolymer exhibits crystalline melting between 30 and 40 °C, where the storage modulus drops from approximately 10^7^ Pa to 10^4^ Pa, indicating a stiff-to-soft transition. Under dry conditions, this composition shows an adhesion strength of 1.67 MPa to glass, which remains 1.2 MPa underwater, and it can support a hanging load of 5 kg. The copolymer adheres well to glass and aluminum but shows weak adhesion to PTFE. After surface abrasion, the adhesion strength to glass increases to 1.6–1.8 MPa. In summary, the copolymer achieves effective underwater adhesion through the synergy of hydrophobic water displacement, thermally induced stiff-to-soft switching, and hydrogen bonding.

## 1. Introduction

Achieving strong adhesion in humid environments or under fully submerged conditions has long been a technical challenge in fields such as medical implants [1], marine engineering [2], underwater repair [3], and daily waterproof bonding [4,5]. Unlike dry interfaces, the primary obstacle to underwater adhesion lies in the preferential formation of a dense hydration layer on the substrate surface by water molecules. Although this water film is only a few nanometers thick, it exerts a strong shielding effect that effectively prevents direct contact between adhesive molecules and the substrate surface [6,7,8]. Even if the adhesive itself possesses highly reactive functional groups, its interfacial bonding capability is greatly compromised if it cannot penetrate or displace this water layer [9,10,11].

Conventional adhesive materials such as cyanoacrylates [12,13], epoxy resins [14,15], and polyurethanes [16,17,18,19] exhibit excellent bonding strength in dry environments, primarily due to their ability to form covalent bonds or strong polar interactions with the substrate [20,21,22,23]. However, once placed underwater or in high-humidity conditions, the performance of these materials often deteriorates rapidly. The reasons are twofold: on the one hand, water molecules competitively bind to the active sites on the substrate surface, displacing the adhesive molecules [24,25]; on the other hand, the inherent instability of some adhesives in water or their sensitivity to moisture during curing further undermines their underwater applicability [26,27,28]. Although strategies such as hydrophobic modification [29,30] or the addition of coupling agents [31,32] have partially alleviated this issue, the long-term reliability of conventional adhesive systems underwater remains unsatisfactory. Inspired by marine organisms such as mussels [33], catechol- or gallol-functionalized polymers have been widely investigated for their ability to penetrate the hydration layer and form strong interactions with various substrates. However, these polyphenolic systems often involve complex synthetic procedures, and their adhesion strength remains significantly lower than that of dry adhesives.

It is worth noting that the success of mussel adhesive proteins relies not only on catechol groups [34] but also on their hydrophobic domains, which play a critical role in displacing interfacial water molecules. Without relying on complex polyphenol synthesis, a rational combination of hydrophobic components and polar groups can also lead to effective underwater adhesion systems.

In this study we copolymerized two structurally well-defined monomers, methacrylic acid (MAA) and stearyl methacrylate (SMA). The long alkyl side chains of SMA combine strong hydrophobicity with reversible crystallinity, enabling a “stiff-to-soft” transition from a semicrystalline state to a viscous flow state near body temperature, thereby displacing the water film and fully wetting the substrate. Meanwhile, the carboxyl groups of MAA form hydrogen bonds with polar surfaces such as glass and metals. The synergy of these two components allows the material to establish stable adhesion even in underwater environments. The experimental results show that at an SMA molar fraction of 85%, the copolymer exhibits a dry adhesion strength to glass of 1.67 MPa, which remains 1.2 MPa underwater, and it can sustain a hanging load of 5 kg.

## 2. Materials and Methods

### 2.1. Materials

Methacrylic acid (MAA, purity > 99%) was purchased from Tokyo Chemical Industry Co., Ltd. (TCI, Tokyo, Japan). Stearyl methacrylate (SMA, purity 96%) and the photoinitiator 2-hydroxy-4′-(2-hydroxyethoxy)-2-methylpropiophenone were purchased from Aladdin Chemical Co., Ltd. (Shanghai, China). All reagents were used as received without further purification.

### 2.2. Synthesis of the Copolymer

**Figure 1:**
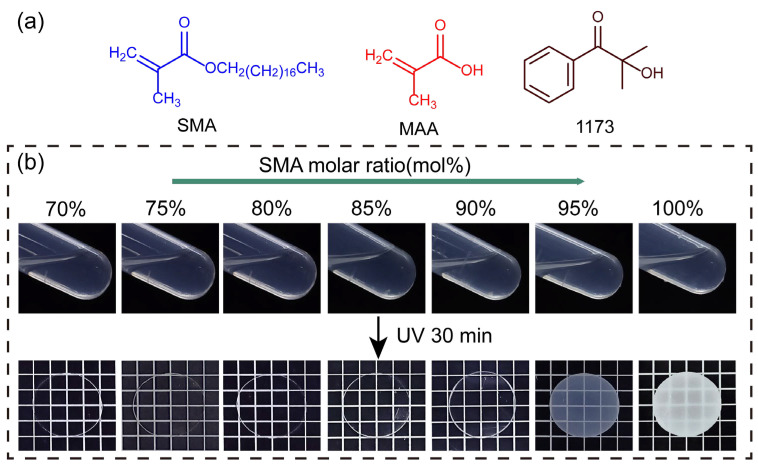
(**a**) Structural formulas of the monomers SMA, MAA, and photoinitiator 1173. (**b**) Photographs of the materials with different molar ratios before and after polymerization.

Methacrylic acid (MAA) and stearyl methacrylate (SMA) were weighed according to the prescribed molar ratio, mixed, and stirred uniformly for 20 min. Subsequently, nitrogen was bubbled through the mixture for 20 min to remove oxygen. The resulting prepolymer solution was then irradiated under a 365 nm UV lamp for 0.5 h to obtain the P(MAA-co-SMA) copolymer (Figure 1). The resulting copolymer is hereinafter referred to as PMS adhesive.

### 2.3. Characterisation Techniques

#### 2.3.1. Tensile Test

Uniaxial tensile tests were conducted using a Sun Technology UTM2103 universal testing machine (Shenzhen, China) equipped with a 100 N load cell. The tests were performed in accordance with the ASTM D-3039 standard at a constant strain rate of 0.1 s^−1^. The tested materials were copolymers of methacrylic acid and stearyl methacrylate at various molar ratios. To ensure statistical reliability, six parallel specimens were tested for each composition, and the results are reported as mean values. The average Young’s modulus and toughness were calculated from the stress–strain curves to compare the mechanical properties of the copolymers with different molar ratios.

#### 2.3.2. Lap-Shear Tests

Lap shear tests were conducted using a universal testing machine (UTM2103, Shenzhen Sun Technology, Shenzhen, China) at a crosshead speed of 20 mm/min. The adhesion area was 1 cm × 1 cm. After bonding, the specimens were heated with a heat gun, fixed with clamps, and subjected to an applied pressure of approximately 200 kPa. At least three replicate measurements were performed for each test condition, and the results are presented as mean ± standard deviation. All lap shear failures exhibited adhesive failure. Stainless steel (SUS) was also used as one of the test substrates.

#### 2.3.3. Fourier Transform Infrared Spectroscopy Characterization (FTIR)

The chemical structure of the samples was characterized using a Fourier transform infrared spectrometer (Nicolet-iS50, Thermo Fisher Scientific, Waltham, MA, USA). The samples were prepared as uniform thin films and sandwiched between two zinc selenide (ZnSe) windows. Infrared spectra were collected in transmission mode over a wavenumber range of 4000–400 cm^−1^ at a resolution of 4 cm^−1^, with 32 scans per spectrum.

#### 2.3.4. Rheological Characterization

Temperature sweep measurements of the adhesives were performed using a HAAKE MARS 60 advanced rotational rheometer (Thermo Fisher Scientific, Karlsruhe, Germany) equipped with parallel plate fixtures (8 mm diameter). The tests were conducted in oscillatory mode over a temperature range from −10 °C to 80 °C, with a strain amplitude of 0.01% and an oscillation frequency of 1 Hz.

#### 2.3.5. Differential Scanning Calorimetry (DSC)

The melting behavior of the samples was characterized using a differential scanning calorimeter (DSC, TA Q250, New Castle, DE, USA) under a nitrogen atmosphere. The heating rate was 10 °C·min^−1^, and the temperature scanning range was from −20 °C to 70 °C. The test procedure consisted of two cycles of heating–cooling–reheating. The first heating ramp was used to erase the thermal history of the samples, and the melting temperature was determined from the second heating curve.

#### 2.3.6. Low-Field Nuclear Magnetic Resonance (NMR) Characterization

Low-field NMR measurements were performed on the copolymer adhesives with different molar ratios using a low-field NMR spectrometer. The samples were placed in NMR glass tubes, and the measurement temperature was maintained at 25 °C. Variable-temperature tests were conducted using a temperature control accessory integrated with the NMR system. One-dimensional low-field NMR measurements were carried out using the Carr–Purcell–Meiboom–Gill (CPMG) pulse sequence.

## 3. Results

### 3.1. FTIR Analysis

**Figure 2:**
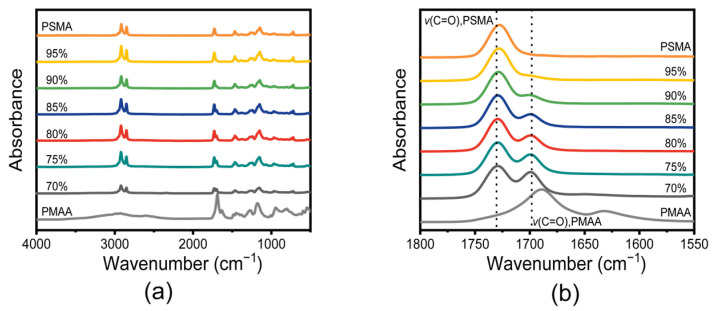
(**a**) FTIR spectrum of PMS adhesives in the region of 4000–500 cm^−1^. (**b**) FTIR spectrum of PMS adhesives in the region of 1800–1550 cm^−1^.

First, FTIR spectroscopy was employed to verify the successful copolymerization of AA and SMA (Figure 2a). As shown in Figure 2b, further analysis of the C=O stretching region reveals hydrogen bonding interactions between carboxyl and ester groups. The C=O peak of PMAA appears at ~1700 cm^−1^ and shifts to a higher wavenumber after copolymerization, with a relatively broad peak shape, indicating the formation of COOH···O=C hydrogen bonds. With increasing SMA content, the PSMA C=O peak (originally at ~1730 cm^−1^) shifts to a lower wavenumber with a slight increase in intensity, while the PMAA C=O peak shifts to a higher wavenumber with a gradual decrease in intensity until it disappears for pure PSMA. These trends further confirm the presence of hydrogen bonding between the carboxyl and ester groups in the copolymer. Furthermore, the intensity of the C–H stretching vibration peaks of the long alkyl chains (2920 and 2850 cm^−1^) significantly increased with increasing SMA ratio. These results demonstrate that the copolymer simultaneously retains hydrophobic long chains and carboxyl groups capable of forming hydrogen bonds, providing a structural basis for subsequent underwater adhesion.

### 3.2. Low-Field NMR Analysis

**Figure 3:**
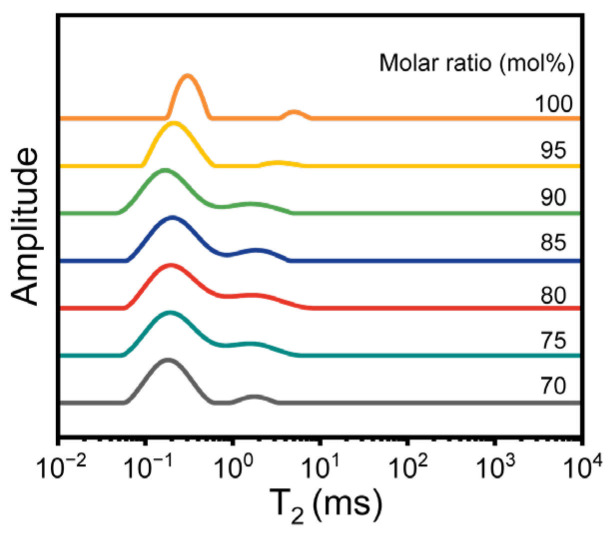
Low-field ^1^H Nuclear Magnetic Resonance Spectra of PMS adhesives.

Low-field ^1^H NMR spectroscopy was employed to probe the mobility of different protons in the PMS adhesive, revealing the compositional dependence of the copolymer structure at the molecular motion level (Figure 3). With increasing SMA content, the transverse relaxation time (T_2_) of the alkyl protons exhibited a distinct bicomponent feature: one component exhibited fast relaxation, corresponding to the rigid regions with restricted chain segment motion; the other component exhibited slower relaxation, corresponding to the more mobile chain segments, primarily the terminal portions of the long alkyl side chains. When the SMA molar fraction reached 85%, the proportion of the slow-relaxation component increased significantly. This indicates that the long alkyl side chains possess a high degree of freedom, allowing the molecular chains to maintain considerable mobility even within a confined space. Such mobility facilitates the spreading of the adhesive on the substrate surface, thereby increasing the effective contact area. Meanwhile, the relaxation of the carboxyl protons was faster than that of the alkyl protons, primarily due to the formation of hydrogen bonds between carboxyl groups or between carboxyl groups and the substrate, which restricted the motion of the carboxyl protons and accelerated their relaxation. Collectively, the low-field NMR results verify, from the perspective of molecular motion, the mobility of the hydrophobic long chains and the hydrogen-bonding behavior of the carboxyl groups, providing a basis for understanding the relationship between the microstructure and macroscopic adhesion performance of the PMS adhesive.

### 3.3. DSC Analysis

**Figure 4:**
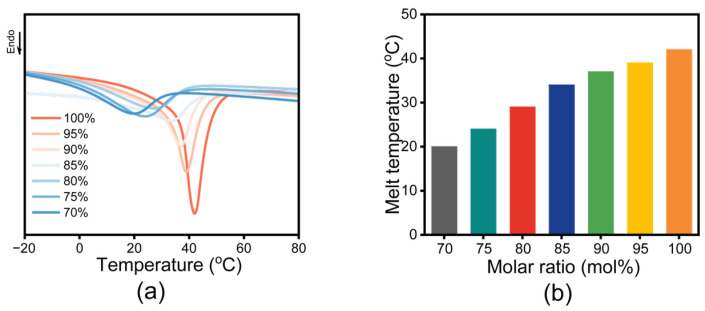
(**a**) DSC heating curve of PMS adhesives. (**b**) Melting temperature summarized from the DSC heating curves of PMS adhesives.

The crystallization behavior and stiff-to-soft switching capability of the PMS adhesives were verified by DSC measurements. As shown in Figure 4a,b, the pure SMA homopolymer exhibits a distinct crystalline melting peak at approximately 42 °C. With the introduction of MAA, the melting point of the PMS adhesives gradually decreases. When the SMA molar fraction reaches 85%, the melting temperature decreases to approximately 35 °C, close to body temperature, which endows the adhesive with broader application potential in scenarios such as biomedicine. The crystalline phase melts near body temperature, enabling a “stiff-to-soft” transition from a semicrystalline state to an amorphous state. In the crystalline state, the material is relatively stiff, facilitating handling and shaping; after melting, the molecular chain mobility increases, promoting wetting of the adhesive interface. The copolymer has a linear structure; the long alkyl side chains of SMA can form crystalline domains that serve as physical crosslinking points (physical crosslinking network), and the carboxyl groups provided by MAA are responsible for interfacial hydrogen bonding with the substrate. Subsequent experiments were all conducted using the PMS adhesive with an SMA content of 85 mol%. The composition with 85 mol% SMA was selected because it balances three key contributions: the hydrophobic long alkyl chains of SMA provide water-displacement ability and crystalline cohesive strength, while the carboxyl groups of MAA offer interfacial hydrogen-bonding sites. At this ratio, the melting temperature of the crystalline domains is approximately 35 °C, close to body temperature, which facilitates thermally induced stiff-to-soft switching and interfacial wetting.

### 3.4. Mechanical Properties Analysis

**Figure 5:**
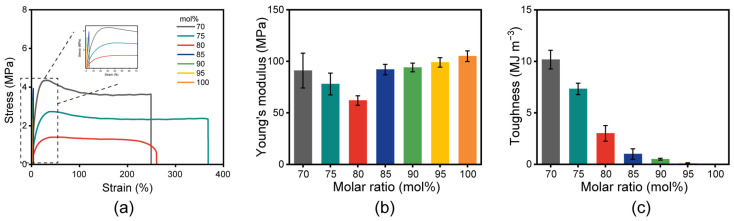
(**a**) Tensile mechanical curves of PMS adhesives with different molar ratios. (**b**) The calculated Young’s modulus of PMS adhesives. (**c**) The calculated toughness of PMS adhesives.

To investigate the effect of different molar ratios on the mechanical properties of the PMS adhesive, uniaxial tensile tests were conducted on a series of samples at room temperature. The representative stress–strain curves, along with the calculated Young’s modulus and toughness, are shown in Figure 5.

Figure 5a presents the tensile stress–strain curves of the samples with different molar ratios. It can be observed that all samples exhibit a distinct elastic deformation stage followed by a subsequent plastic plateau stage, without obvious brittle fracture characteristics, indicating that the materials generally display ductile fracture behavior. As the molar ratio varies, the mechanical response of the samples shows significant differences. The sample with a molar ratio of 70 mol% exhibits the highest initial modulus and yield strength. After reaching the peak stress, the stress decreases slowly, followed by a long plateau stage, with an elongation at break exceeding 350%, demonstrating a combination of relatively high strength and good ductility. As the molar ratio further increases, both the yield strength and the plateau stress gradually decrease. For example, the sample with 80 mol% exhibits a maximum stress of approximately 4 MPa, and the elongation at break decreases to below 50%. When the molar ratio increases to 90 mol% or higher, the stress level of the samples decreases significantly, the stress plateau before fracture is markedly shortened, and both the overall strength and elongation deteriorate substantially, exhibiting softer and more fracture-prone mechanical characteristics.

Figure 5b shows the statistical results of Young’s modulus for samples with different molar ratios. At a molar ratio of 70 mol%, the Young’s modulus is approximately 90 MPa. As the molar ratio increases from 70 mol% to 80 mol%, the modulus decreases significantly, reaching a minimum of approximately 60 MPa, which may be related to changes in the physical crosslinking network (crystalline domains of SMA side chains serving as physical crosslinks) and increased chain flexibility. When the molar ratio continues to increase from 80 mol% to 100 mol%, the Young’s modulus shows a continuous upward trend, reaching approximately 105 MPa at 100 mol%, exhibiting a “decrease first, then increase” pattern. This phenomenon suggests that in the low molar ratio range, as the SMA content increases, the decrease in crosslinking density or chain rigidity leads to a reduction in modulus. When the molar ratio exceeds 80 mol%, structural changes within the material, such as increased crystallinity, enhanced phase separation, or increased hard segment content, increase the material’s stiffness, leading to a subsequent increase in modulus.

In contrast to the trend in modulus, the toughness of the material exhibits a continuous decrease with increasing molar ratio, as shown in Figure 5c. The sample with a molar ratio of 70 mol% shows the highest toughness, approximately 10 MJ·m^−3^, demonstrating excellent energy absorption capacity. As the molar ratio gradually increases to 75 mol% and 80 mol%, the toughness decreases to approximately 7.5 MJ·m^−3^ and 3 MJ·m^−3^, respectively. When the molar ratio exceeds 85 mol%, the toughness of the material decays sharply, approaching zero for samples with molar ratios above 90 mol%, indicating that the material’s impact resistance and fracture resistance are significantly deteriorated under high molar ratio conditions, making it prone to brittle fracture under external force. Combined with the stress–strain curves, the continuous decrease in toughness can be attributed to two main factors: on the one hand, the elongation at break decreases significantly with increasing molar ratio, reducing the plastic deformation that can occur during stretching; on the other hand, the overall stress level of the material decreases markedly, and the area under the stress–strain curve diminishes accordingly, ultimately leading to a sustained decrease in toughness.

### 3.5. Rheological Analysis

**Figure 6:**
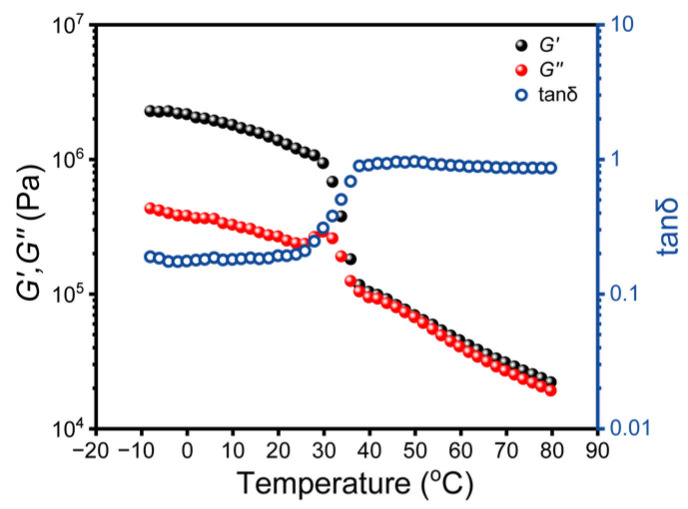
Rheological heating curves of the PMS adhesive.

To further explore the dynamic mechanical behavior and rheological properties of the PMS adhesive over a wide temperature range, we performed temperature-sweep rheological tests and systematically characterized the temperature dependence of its storage modulus (G′), loss modulus (G″), and loss factor (tanδ). The results are presented in Figure 6.

From the overall trend, the rheological behavior of the PMS adhesive can be divided into two distinct stages based on temperature. In the low-temperature region (approximately −10 to 35 °C), G′ remains higher than G″ and stays on the order of 10^5^–10^6^ Pa, indicating typical solid-like elastic behavior. As the temperature gradually increases, both G′ and G″ decline slowly, which is mainly attributed to enhanced polymer chain segment motion and a consequent reduction in material rigidity. Throughout this stage, tanδ remains between 0.1 and 1 and changes only mildly, suggesting weak viscous dissipation, dominance of elastic response, and an overall state corresponding to a stable glassy or early rubbery regime.

When the temperature rises into the 30–40 °C range, the decreases in G′ and G″ accelerate markedly, and the two curves cross each other, giving rise to a clear rheological transition point. Beyond this crossing, G″ gradually exceeds G′, marking a shift from elasticity-dominated to viscosity-dominated behavior. In this interval, tanδ rises sharply, reaches a peak quickly, and then levels off. This process corresponds to the glass transition or the transition from the rubbery state to the viscous flow state: elevated temperature provides polymer chain segments with sufficient energy to overcome interchain interactions, thereby greatly enhancing chain slippage and flow capability, with viscous behavior gradually taking over. In the high-temperature region (above 40 °C), both G′ and G″ continue to decrease with temperature, and their decreasing trends become parallel, while tanδ stabilizes at a value close to unity, indicating that the material has entered a stable viscous flow state where elastic and viscous responses are relatively balanced. This rheological behavior demonstrates that at elevated temperatures, chain segment motion in the PMS adhesive is fully activated, and the material exhibits good flowability and deformation capacity.

### 3.6. Dry Adhesion Analysis

**Figure 7:**
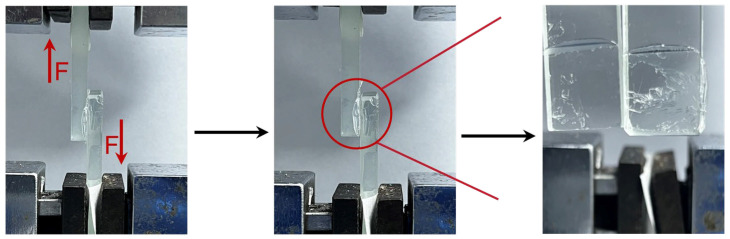
Photographs of the adhesion tests.

In the shear test, the adhesion process of the adhesive is correlated with its crystalline-melting behavior (Figure 7). The sample was first heated above the crystalline melting temperature, where the long alkyl side chains transitioned from an ordered arrangement to disordered motion. As a result, the material became soft and flowable, allowing it to fully spread over the glass surface. Subsequently, pressure was applied and the sample was cooled, during which the polymer chains recrystallized and solidified, restoring the cohesive strength and completing the fixing process. During the subsequent debonding process, the failure mode was characterized as brittle fracture rather than ductile failure by viscous pull-out.

**Figure 8:**
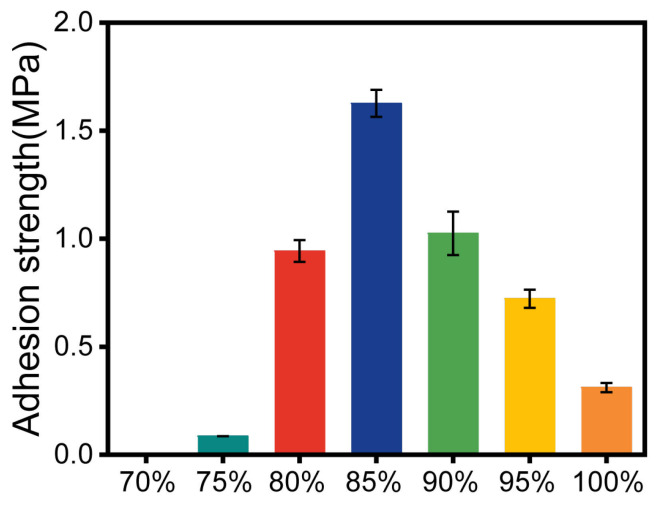
Dry adhesion strength of PMS adhesives.

Subsequently, shear adhesion tests were conducted on PMS adhesives with different molar ratios under dry conditions, and the results are shown in Figure 8. When the SMA molar fraction increased from 70% to 85%, the adhesion strength gradually increased, reaching a maximum value of 1.67 MPa at 85%. Further increasing the SMA content to 100% led to a decrease in adhesion strength. Under dry conditions, without the interference of water molecules, the measured adhesion strength primarily reflects the intrinsic interfacial bonding capability and cohesive strength of the material. In this system, an appropriate amount of MAA provides carboxyl groups capable of forming hydrogen bonds or polar interactions with the glass surface, while the long alkyl side chains of SMA impart hydrophobicity and cohesive toughness to the material. The optimal balance between these two components is achieved at a ratio of 85:15 (SMA:MAA), which ensures sufficient interfacial binding sites while maintaining good cohesive strength, thereby yielding the best adhesion performance.

### 3.7. Underwater Adhesion Analysis

**Figure 9:**
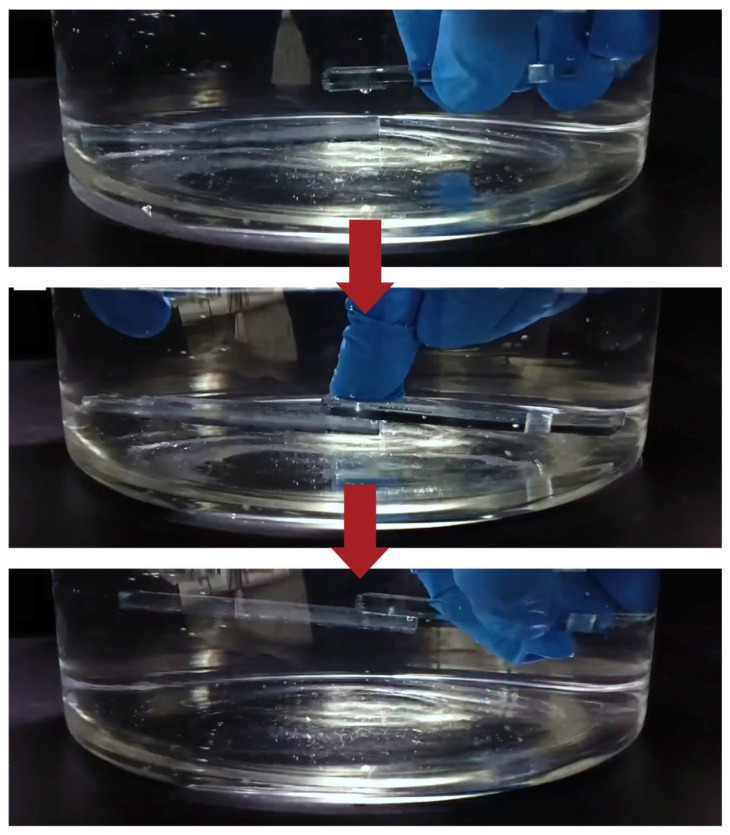
Demonstration of underwater adhesion performance.

Figure 9 illustrates the specific process of underwater adhesion operation using this adhesive. The adhesive was first applied to one end of a glass slide, which was then fully immersed in 40 °C water. The bonding was subsequently performed with another glass slide entirely under water. It can be observed that a strong and robust bonding was rapidly established between the two glass slides, and the adhesive exhibited stable and strong adhesion even after prolonged underwater immersion.

The origin of this underwater adhesion capability is primarily attributed to the strong hydrophobicity of the long alkyl side chains of SMA in the copolymer. The major challenge in underwater adhesion is that water molecules tend to penetrate the gap between the adhesive and the substrate along the interface, disrupting effective contact and ultimately leading to bonding failure. In this system, the hydrophobic long chains actively displace water molecules near the interface during the bonding process, effectively acting as a barrier between water and the adhesive interface. This barrier prevents further diffusion of water into the adhesive layer. Even when the entire testing environment is surrounded by water, the adhesive interface maintains a relatively dry contact state, thereby retaining most of its adhesion strength. Consequently, this adhesive achieves stable and durable bonding even in an underwater environment at 40 °C.

**Figure 10:**
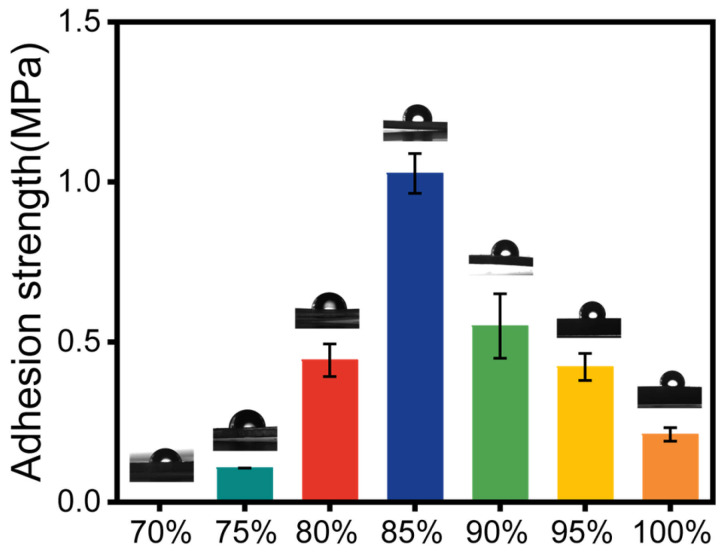
Underwater adhesion strength and corresponding contact angle measurements of the PMS adhesive.

Underwater adhesion strength tests were conducted using the same single-lap shear method as employed for dry conditions. Figure 10 summarizes the underwater adhesion strength data of the samples with different molar ratios. Overall, the measured values underwater were lower than their corresponding dry-state values, indicating that the presence of water molecules inevitably interferes to some extent with interfacial bonding. Notably, the trend of underwater adhesion strength as a function of SMA content was consistent with that observed under dry conditions: as the SMA molar fraction increased from 70 mol% to 85 mol%, the adhesion strength gradually increased, reaching a maximum value of approximately 1.2 MPa at 85 mol%; further increasing the SMA content to 100 mol% led to a decrease in adhesion strength. This result indicates that the 85:15 molar ratio also represents the optimal composition for underwater adhesion. We also performed contact angle measurements at different ratios. As the SMA content increased, the hydrophobicity of the sample was enhanced, endowing the PMS adhesive with excellent water displacement capability.

**Figure 11:**
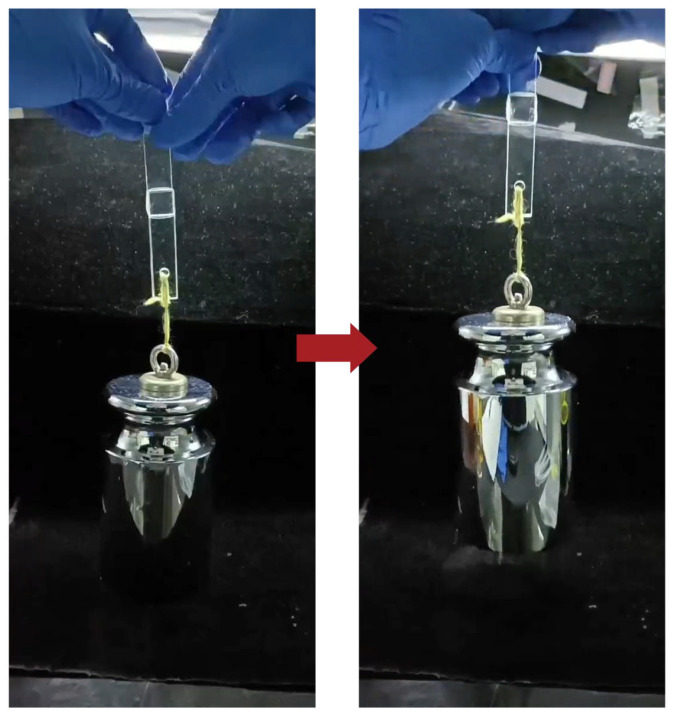
Demonstration of underwater adhesion strength under pulling conditions.

The lifting experiment shown in Figure 11 further validated the load-bearing capacity of this composition under practical conditions. In the experiment, the bonded specimen was suspended with a 5 kg weight to observe whether debonding or slippage occurred. The PMS adhesive was used at a proportion of 85%. The results showed that the adhesive system could stably bear this load without debonding, indicating that its adhesion strength has reached a practical level. A load-bearing capacity of 5 kg provides a sufficient safety margin for most underwater bonding scenarios. Therefore, this adhesive not only exhibits favorable adhesion data in laboratory tests but also demonstrates reliable performance in practical load-bearing applications.

**Figure 12:**
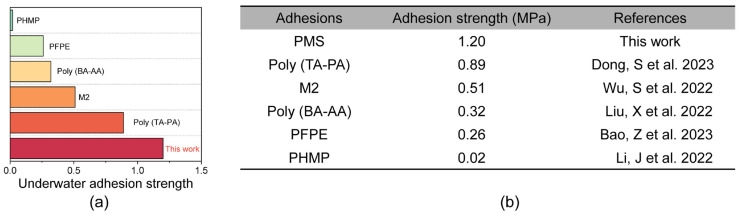
(**a**) Comparison of underwater adhesion performance. (**b**) Data for comparison of underwater adhesion performance [35,36,37,38,39].

We compared the underwater adhesion strength of the PMS adhesive with typical underwater adhesive systems reported in the literature [35,36,37,38,39]. As shown in Figure 12, the PMS adhesive exhibits an adhesion strength of approximately 1.2 MPa to glass substrates under underwater conditions. This performance is comparable to or even exceeds that of many existing systems, rendering it promising for a wide range of applications in underwater operations.

**Figure 13:**
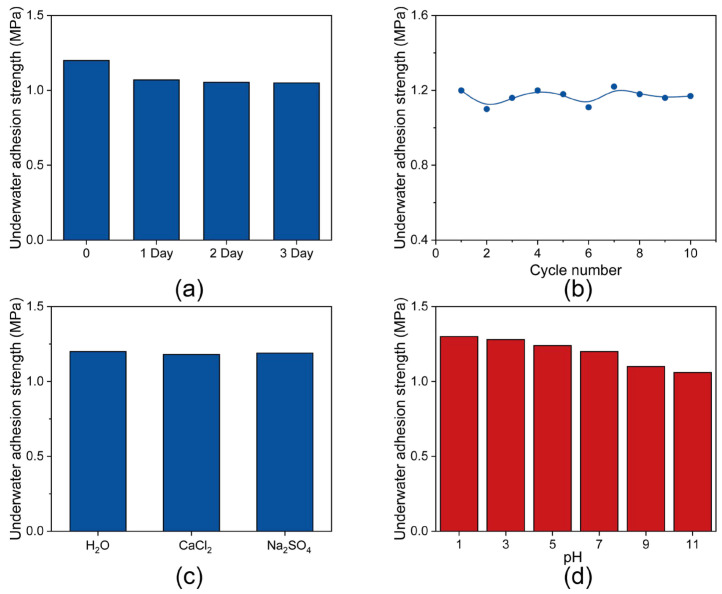
(**a**) Underwater adhesion strength after exposure to aqueous media for different numbers of days. (**b**) Adhesion strength under repeated wet-dry cycles. (**c**) Underwater adhesion strength in different salt solutions. (**d**) Underwater adhesion strength in solutions with different pH values.

To evaluate long-term durability, the PMS adhesive (85 mol% SMA) was subjected to extended aqueous immersion, repeated wet–dry cycles, saline solutions, and varying pH conditions. Owing to the strong hydrophobicity of the SMA side chains, the adhesion strength decreased only slightly with prolonged immersion. As shown in Figure 13, the adhesive remained stable over multiple wet–dry cycles, indicating good reversibility of the crystalline physical crosslinking network. Salt solutions (CaCl_2_ and Na_2_SO_4_) had little effect on adhesion, as the hydrophobic chains effectively shield ionic interference. Under acidic conditions, adhesion strength increased slightly due to protonated carboxyl groups (-COOH) favoring hydrogen bonding, whereas under alkaline conditions, partial deprotonation to -COO^−^ led to a modest decrease. Overall, the PMS adhesive exhibits not only strong short-term underwater adhesion but also good long-term durability and environmental robustness.

### 3.8. Substrate Universality of Underwater Adhesion

**Figure 14:**
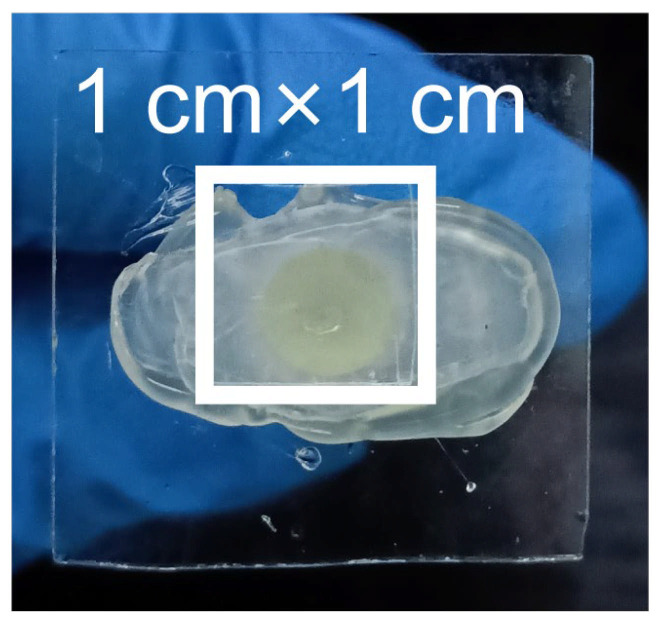
Photograph of the adhesion area.

As shown in Figure 14, the adhesion area of the adhesive was 1 cm × 1 cm. All specimens were bonded and tested underwater to simulate practical applications in humid or submerged environments.

**Figure 15:**
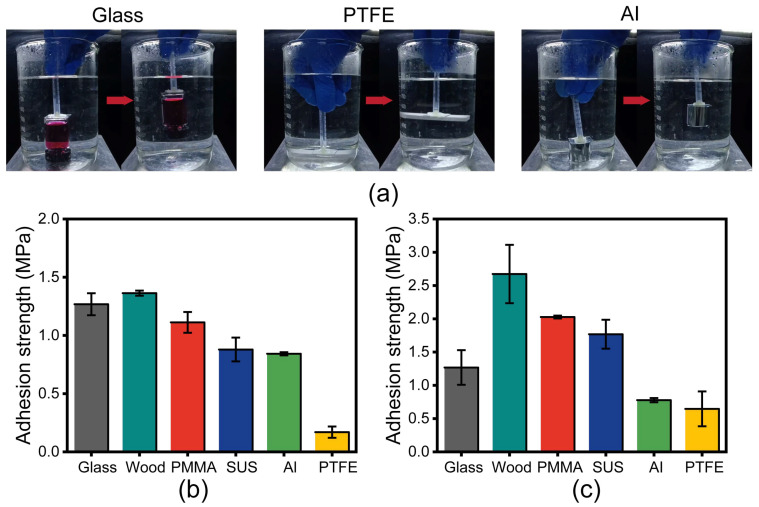
(**a**) Photographs of adhesion on glass, PTFE, and aluminum substrates. (**b**) Underwater adhesion strength on different substrates. (**c**) Underwater adhesion strength on different substrates after sandpaper abrasion.

Figure 15a illustrates the adhesion behavior of the PMS adhesive on three typical substrates—glass; polytetrafluoroethylene (PTFE); and aluminum—underwater. The PMS adhesive was used at a proportion of 85%. It can be clearly observed that the adhesive exhibits significantly different adhesion effects on different substrates in the underwater environment. On glass and aluminum surfaces, the adhesive adheres well and forms a relatively strong bond. In contrast, on the PTFE surface, the spreading and adhesion effects are relatively weak. This indicates that the adhesive does not perform equally well on all substrates; its adhesion performance is closely related to the surface properties of the substrate. Nevertheless, aside from PTFE, which is a typical low-surface-energy nonpolar material, the adhesive exhibits good underwater adhesion to common polar substrates such as glass and aluminum, demonstrating a certain degree of substrate adaptability.

Figure 15b summarizes the underwater adhesion strengths of the PMS adhesive on three typical substrates. As clearly shown by the data, the adhesion strength follows a distinct order: highest on glass (~1.2 MPa), intermediate on aluminum (~0.9 MPa), and lowest on PTFE (~0.3 MPa). This order is not coincidental but is closely related to the surface polarity of the substrates and the density of active sites available for interfacial interactions.

The glass surface is rich in silanol groups (Si–OH), which can form hydrogen bonds with the carboxyl groups of MAA in the copolymer. Moreover, the high density of these active sites enables the strongest interfacial bonding. The aluminum surface, although covered with a natural oxide layer (Al_2_O_3_) that imparts a certain degree of polarity and can engage in acid–base or coordination interactions with carboxyl groups, typically has a lower density of surface hydroxyl groups than glass, resulting in a slightly lower adhesion strength. PTFE, in contrast, represents a completely different case. As a typical low-surface-energy, nonpolar material, it lacks functional groups capable of forming hydrogen bonds or polar interactions with carboxyl groups. On such a substrate, the adhesive can only rely on physical attachment through its hydrophobic nature, making it difficult to establish sufficiently strong interfacial bonding. Consequently, the adhesion strength on PTFE is significantly lower. This adhesive is more suitable for underwater bonding of polar substrates such as glass and metals, while its effectiveness on inert materials like PTFE is limited.

After abrasion with 80-grit sandpaper, the underwater adhesion strength on all substrates increased to varying extents (Figure 15c). Following abrasion, the adhesion strength on glass increased to approximately 1.6 MPa, on aluminum to approximately 1.3 MPa, and even on PTFE, it showed a modest increase to approximately 0.5 MPa. Abrasion increases surface roughness and the effective contact area, while potentially introducing fresh surfaces and localized polar sites (especially on metals and glass), thereby enhancing mechanical interlocking and interfacial bonding. Nevertheless, even after abrasion, the adhesion strength on PTFE remained significantly lower than that on glass and aluminum, indicating that the chemical inertness of PTFE remains the dominant limiting factor, and physical roughening alone cannot fully compensate for the lack of interfacial chemical interactions.

**Figure 16:**
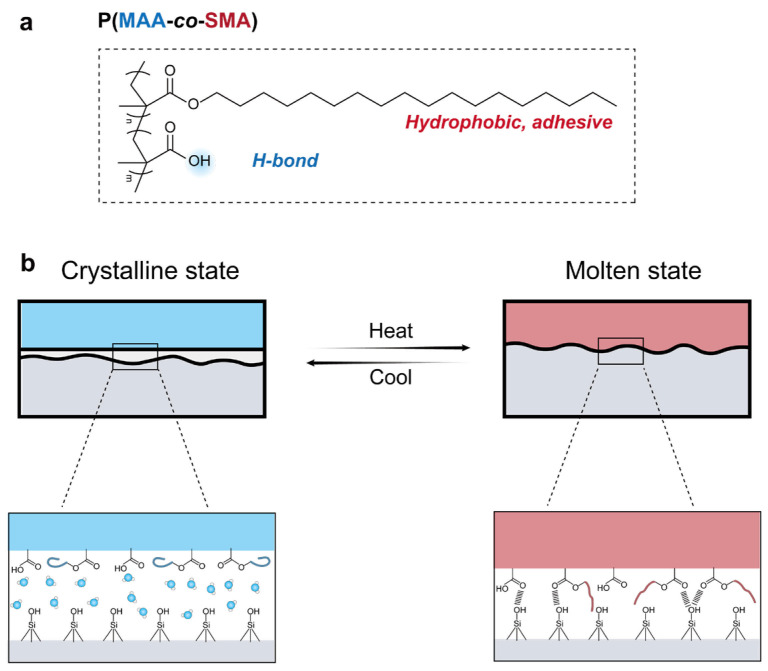
(**a**) Structural formula of SMA and MAA copolymer. (**b**) Underwater adhesion mechanism of PMS adhesive.

The adhesion performance of the PMS adhesive in underwater environments is governed not by a single factor but by the synergistic combination of hydrophobic water displacement, thermally induced stiff-to-soft switching, and interfacial bonding. The primary obstacle to underwater adhesion is the ubiquitous hydration layer on substrate surfaces. Without effective displacement of this water film, direct contact between adhesive molecules and the substrate is difficult to achieve. The long alkyl side chains of SMA in PMS are strongly hydrophobic, actively displacing water molecules near the interface during the bonding process and forming a hydrophobic barrier. This barrier not only reduces water diffusion into the adhesive layer but also creates a relatively dry local environment for subsequent interfacial bonding (Figure 16).

Simultaneously, the thermally induced stiff-to-soft switching plays a critical role. When heated above the crystalline melting temperature, the copolymer transitions from a semicrystalline state to an amorphous state, becoming soft and significantly increasing molecular chain mobility. This allows better conformal contact with microscopic surface irregularities of the substrate, thereby increasing the effective contact area. After bonding, as the temperature decreases, the material recrystallizes and solidifies, restoring its cohesive strength.

At the level of interfacial bonding, both the carboxyl groups of MAA and the carbonyl groups of SMA can form hydrogen bonds with the abundant silanol groups on the glass surface. Among these, the carboxyl groups exhibit stronger hydrogen-bonding capability and serve as the primary source of interfacial interactions. Through the combined action of hydrophobic chains displacing the water film, thermally induced stiff-to-soft switching ensuring conformal contact, and carboxyl/carbonyl groups providing interfacial anchoring, the PMS adhesive achieves stable and robust adhesion even in underwater environments.

## 4. Conclusions

In this study, a series of copolymers of MAA and SMA were prepared, and the variation in composition was found to significantly influence the appearance, mechanical behavior, and adhesion performance of the materials. When the SMA molar fraction exceeded 85%, the material became hard and brittle, whereas the introduction of an appropriate amount of MAA improved its toughness. FTIR spectroscopy confirmed the successful copolymerization of the two monomers, with carboxyl groups and long alkyl side chains coexisting in the polymer system. The DSC and rheological results demonstrated that the copolymer with 85 mol% SMA exhibited a distinct crystalline melting transition at approximately 35 °C, with the storage modulus decreasing from about 10^7^ Pa to 10^5^ Pa, enabling a stiff-to-soft switching near body temperature. This characteristic facilitates substrate wetting during the adhesion process while maintaining adequate initial cohesive strength.

Adhesion tests showed that under dry conditions, the copolymer with 85 mol% SMA achieved an adhesion strength of 1.67 MPa on glass substrates. Under underwater conditions, the same composition retained an adhesion strength of 1.2 MPa and could sustain a hanging load of 5 kg. In terms of substrate adaptability, the copolymer exhibited good adhesion to polar substrates such as glass and aluminum, but weak adhesion to nonpolar PTFE, indicating that the interfacial interactions are dominated by hydrogen bonding and polar forces. Surface abrasion treatment further enhanced the underwater adhesion strength, reaching approximately 1.6 MPa on glass, suggesting that mechanical interlocking from physical roughening can synergistically enhance interfacial chemical bonding.

Overall, the copolymer achieves effective underwater adhesion through the synergistic action of hydrophobic long chains displacing the interfacial water film, carboxyl groups forming hydrogen bonds or polar interactions with the substrate, and a crystalline–melting transition near body temperature. Given its facile synthesis, tunable composition, and mild responsive behavior, this system holds promise for applications in structural bonding in humid environments, underwater repair, and biomedical adhesion.

## Data Availability

The original contributions proposed in this study are included in the article. Further inquiries can be directed to the corresponding author.
